# Supplementary material for: A structural and dynamic visualization of the interaction between MAP7 and microtubules
Source: Nat Commun. 2024 Mar 2;15:1948. doi: 10.1038/s41467-024-46260-5 (PMC10908866; doi:10.1038/s41467-024-46260-5)
Supplement: Supplementary file 1 — Supplementary Information [file 41467_2024_46260_MOESM1_ESM.pdf]

Supplementary Information

A structural and dynamic visualization of the interaction between MAP7 and microtubules

Agnes Adler[1], Mamata Bangera[2], J. Wouter Beugelink[3], Salima Bahri[1], Hugo van Ingen[1], Carolyn A. Moores[2], Marc Baldus[1]

[1]NMR Spectroscopy, Bijvoet Center for Biomolecular Research, Utrecht University, Padualaan 8, 3584 CH Utrecht, The Netherlands

[2]Institute of Structural and Molecular Biology, School of Natural Sciences, Birkbeck, University of London, London WC1E 7HX, UK

[3]Structural Biochemistry, Bijvoet Center for Biomolecular Research, Utrecht University, Padualaan 8, Utrecht, 3584 CH, The Netherlands

Supplementary Figures 1-8

Supplementary Tables 1 and 2

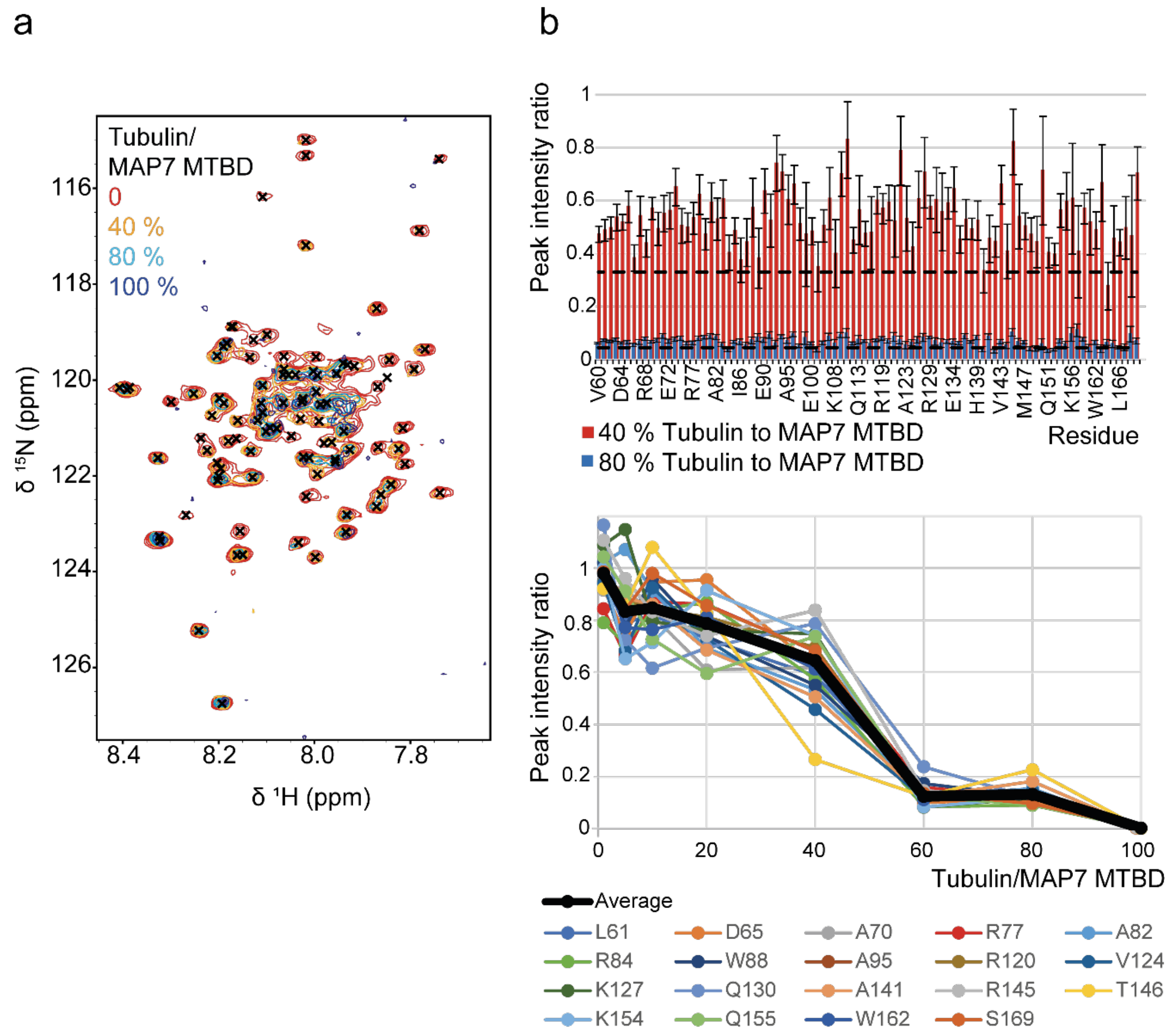

Supplementary Fig. 1: Titration of Taxol-stabilized MT to [^{13}C-^{15}N]- MAP7 MTBD in solution a Overlay of ^{1}H-^{15}N TROSY spectra of varying MT concentrations added to MAP7 MTBD. **b** Intensity ratio over whole MAP7 (top) upon adding 40 % or 80 % polymerized tubulin dimers to MAP7 MTBD. The error bars have been obtained from the signal-to-noise ratio. The black dotted lines are the 10 % trimmed average. Intensity ratio decreases over titration of selected residues (bottom). Errors in peak intensities were based on the spectral noise level obtained from the Sparky software. Source data are provided as a Source Data file.

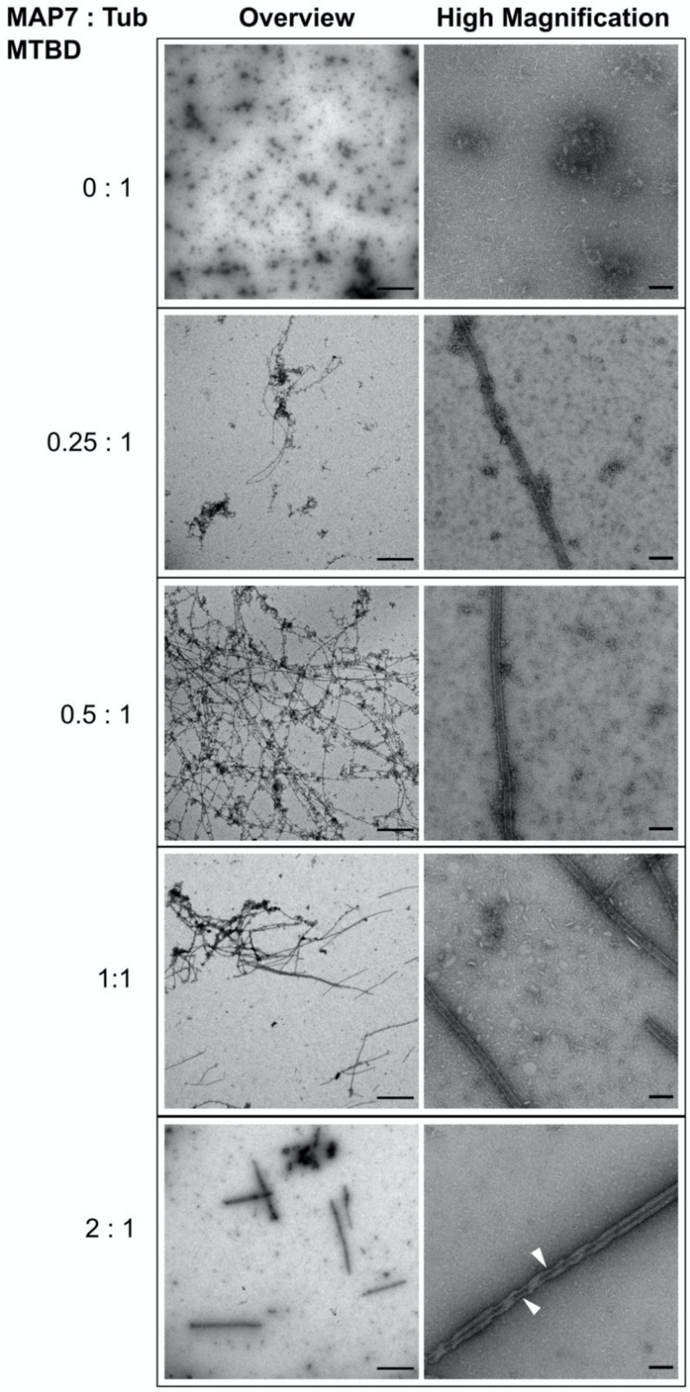

Supplementary Fig. 2: Negative stain EM shows that MAP7 MTBD stabilizes dynamic MTs. Left and right panels show micrographs at low (overview) and high magnifications respectively for MAP7 MTBD and tubulin incubated at molar concentration ratios of 0:1 (no MAP7 MTBD), 0.25:1 , 0.5:1 , 1:1 and 2:1. Concentration of tubulin used is 5 μM. Scale bars represent 2 μm and 100 nm for the left and right panels respectively. White arrows in bottom panel (2:1 MAP7 MTBD: tubulin) show undecorated regions of MT lattice between the thicker surrounding protein (potentially tubulin) layer.

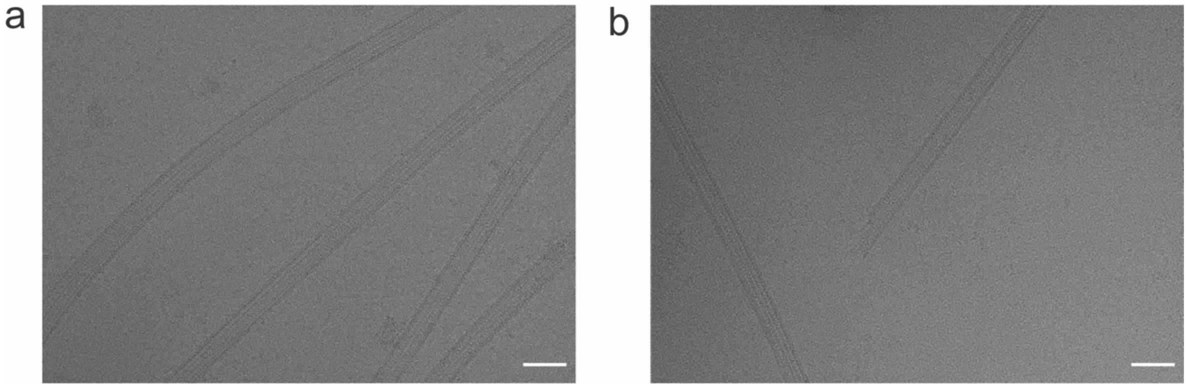

Supplementary Fig. 3: Representative cryo-EM micrographs for a) MAP7 MTBD stabilised MTs and b) Taxol stabilised MTs. Scale bars represents 50 nm.

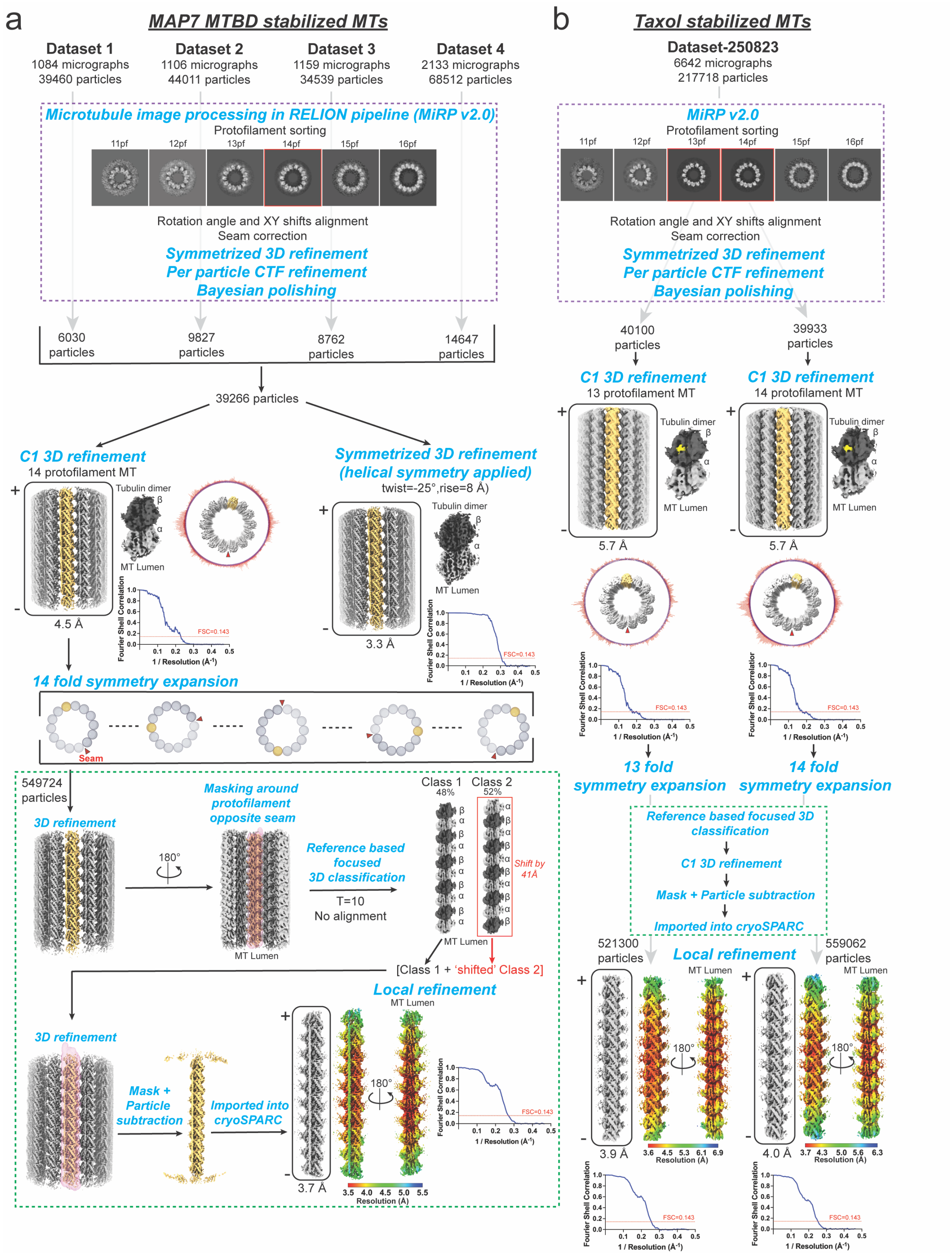

Supplementary Fig. 4: Cryo-EM workflow for a) MAP7 MTBD stabilised MTs and b) Taxol stabilised MTs. Cryo-EM operations are indicated in blue. EM density maps are shown in grey and represent views of the MT from the outside unless stated. Views of the MT from the lumenal side are

indicated as ‘MT Lumen’. For MT reconstructions, the protofilament opposite the seam is shown in yellow. Masks are depicted as transparent surfaces in pink. Round-edge boxes indicate 3D electron density maps that have been deposited to the EMDB. Minus and plus ends of the MT/protofilament are indicated. Histogram plots representing angular distribution of particles used in C1 3D refinement are depicted as a cylinder around the MT (top view) with position of seam indicated by a red arrow and protofilament opposite the seam shown in yellow. Corresponding FSC plots, final maps coloured by local resolution and enlarged densities corresponding to a single tubulin heterodimer have been shown (alpha tubulin: light grey; beta tubulin: dark grey; Taxol: yellow). Source data are provided as a Source Data file.

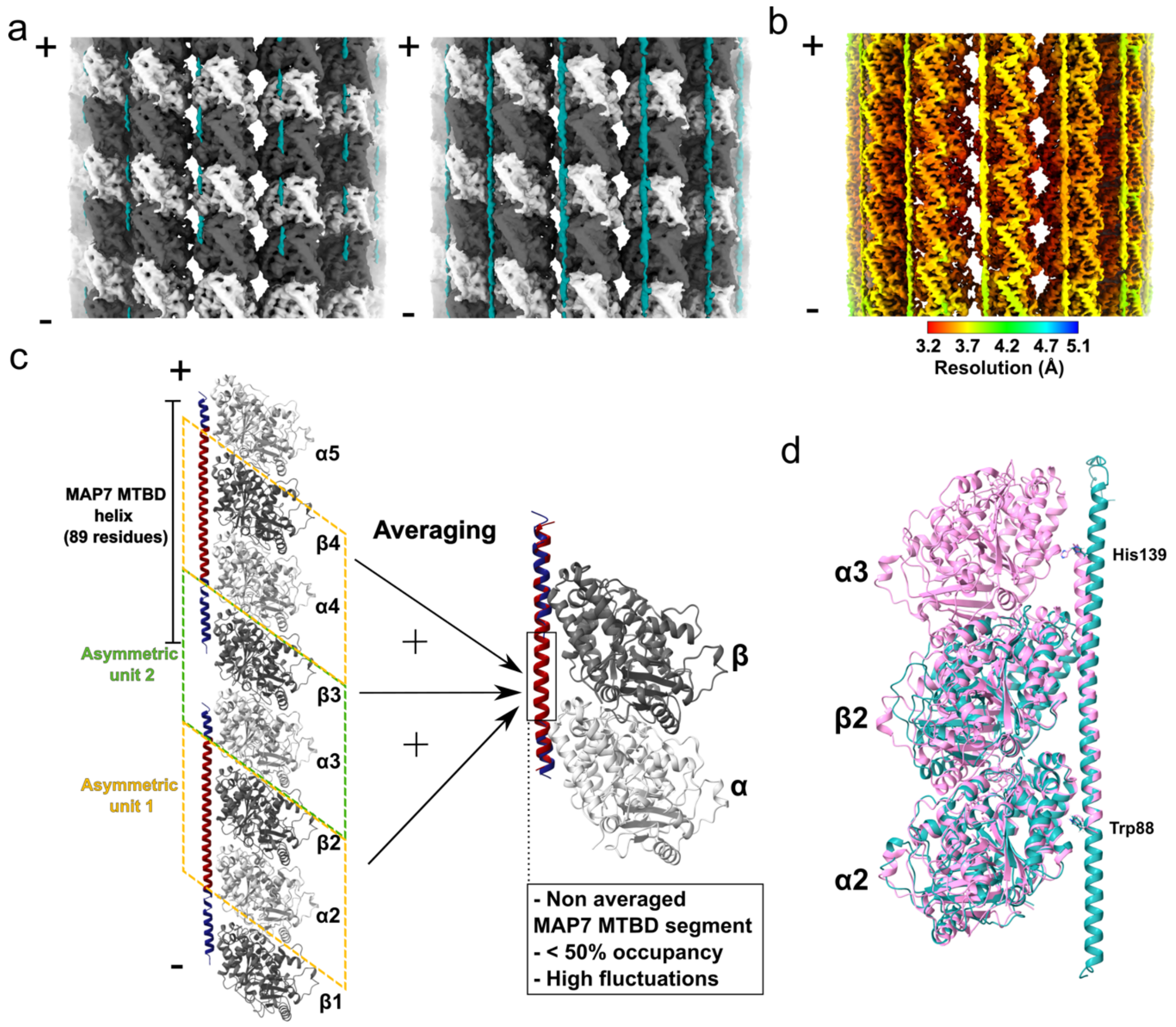

Supplementary Fig. 5: Structure of MAP7 MTBD bound to MTs a Cryo-EM density map (symmetrized reconstructions) of MAP7 MTBD bound MT (1:1 for MTBD and tubulin) when no MAP7 MTBD is added externally at stringent (left) and inclusive (right) map thresholds respectively. Density for the α-tubulin, β-tubulin and MAP7 MTBD is shown in light grey, dark grey and teal respectively. Minus and plus ends of the MT are indicated. **b** Cryo-EM reconstruction (Fig. 2a) coloured based on local resolution as determined by RELION with the colour scheme provided below. Minus and plus ends of the MT are indicated**. c** A single protofilament of MAP7 MTBD bound MT is depicted in cartoon representation. This includes two ribbon models of the entire MAP7 MTBD each bound to four tubulin dimers (PDB ID: 8CR1) positioned along the protofilament. The asymmetric units used in cryo-EM reconstruction are indicated in yellow and green boxes respectively. As has also been shown in Ferro et al. [1] averaging of the asymmetric units as part of the cryo-EM pipeline for MT reconstruction, leads to overlap of heterogenous segments of MAP7 MTBD along the length of its helix and consequently worse resolution for MAP7 MTBD density as compared to the MT. **d** Cartoon representation of model predicted by AlphaFold2 multimer (teal) superposed upon MAP7 MTBD:MT model (pink) previously

determined by cryo-EM (PDB ID: 7SGS). The slight misalignment between these models is because the AlphaFold2 prediction of tubulin heterodimer is in a curved conformation. Bulky residues corresponding to the ends of the 53-residue segment are shown as stick representation in both models and labelled.

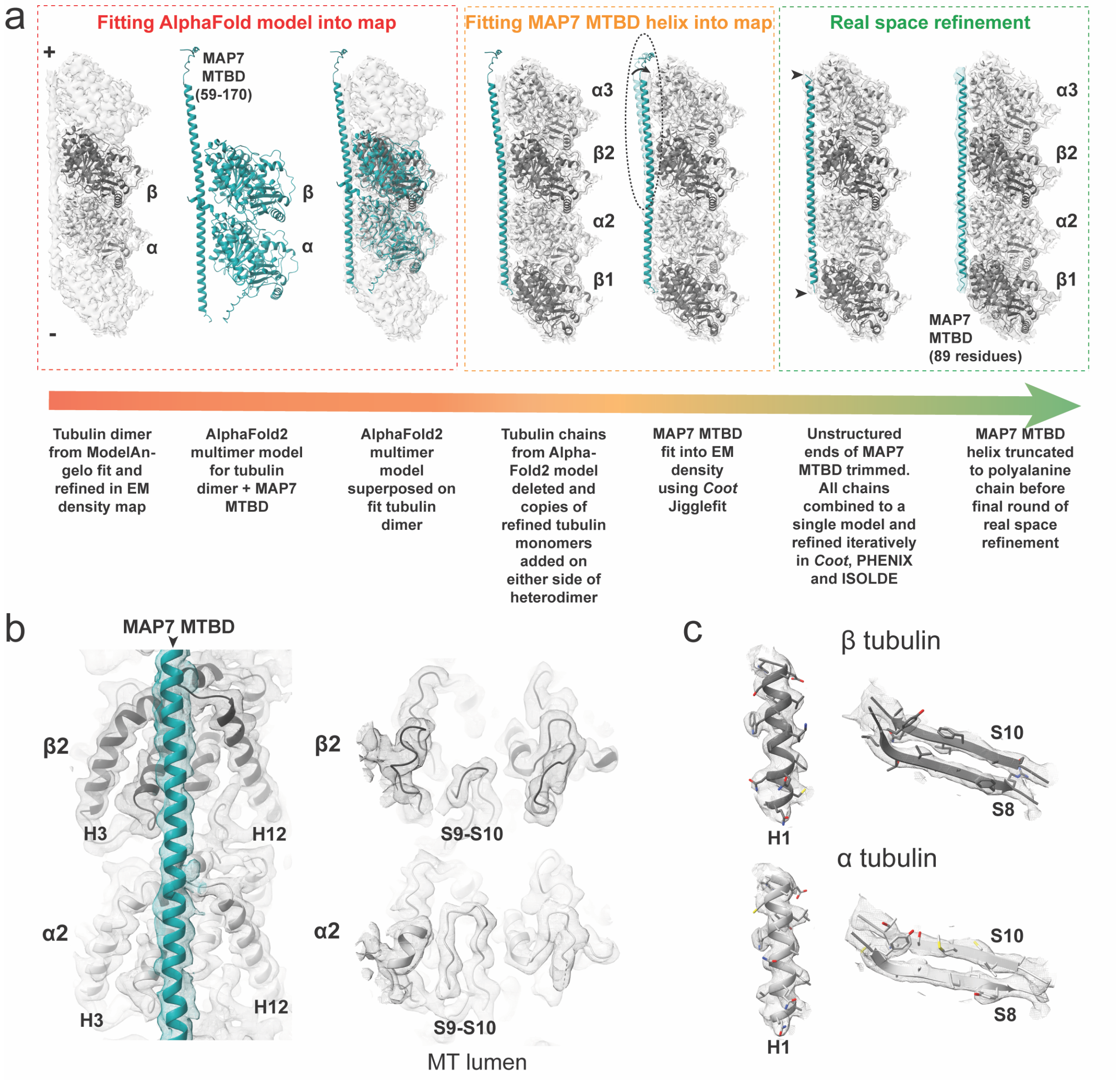

Supplementary Fig. 6: Model building for PDB ID: 8CR1. a The steps in the model building pipeline are depicted from left to right. Cryo-EM density is shown as a grey transparent surface and the models are depicted in cartoon representation (α-tubulin: light grey; β-tubulin: dark grey; MAP7 MTBD: teal). The predicted model obtained from AlphaFold2 multimer is coloured in teal. Changes in the model are indicated with black arrows. **b** Views of the model corresponding to the central tubulin dimer fit in the density map from outside (left) and from the MT lumen (right) are shown with map in mesh and model in cartoon representation. α-tubulin, β-tubulin and MAP7 MTBD are coloured light grey, dark grey and teal respectively. Key structural elements of tubulin are indicated. **c** Representative regions of the map are shown for the final model. The cryo-EM map is shown as a mesh and the model is shown as cartoon with side chains.

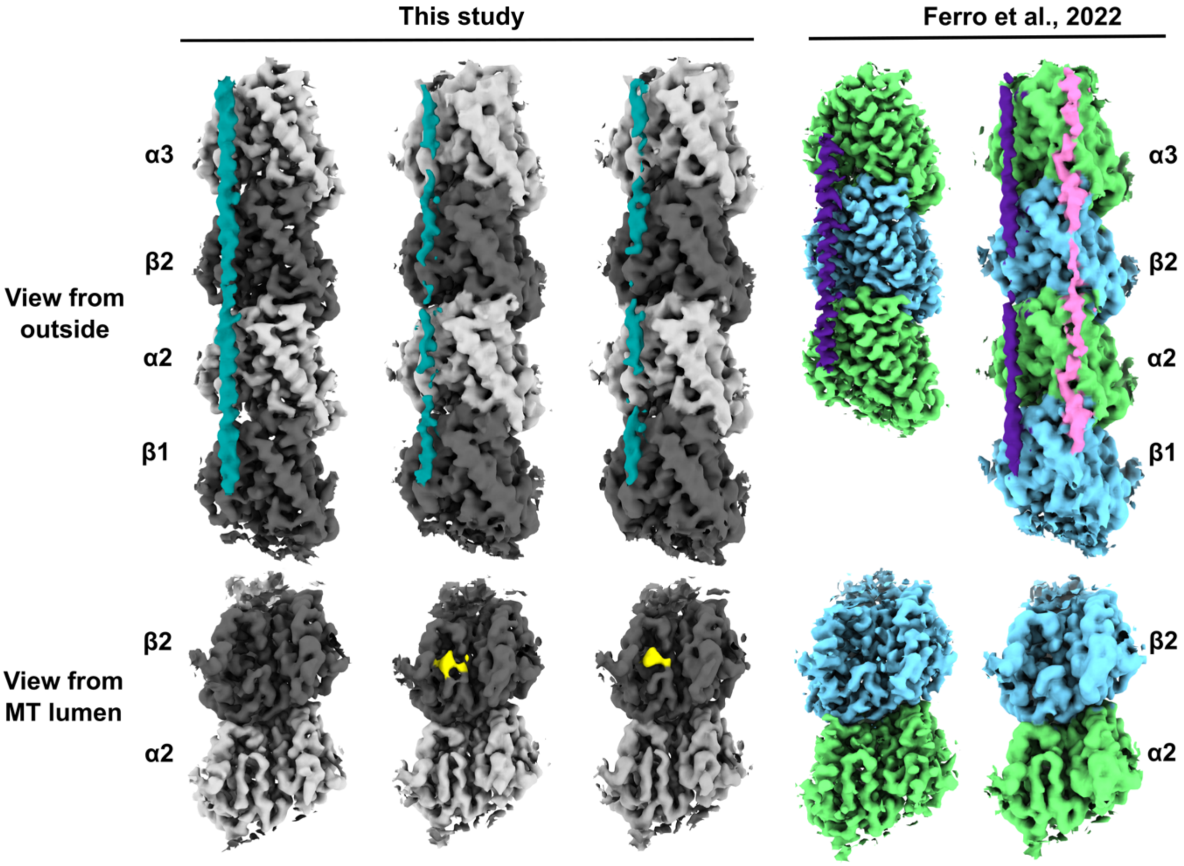

EMDB ID	EMD-19042	EMD-19043	EMD-19044	EMD-25120	EMD-25119
MT architecture	Fragment-14pf	Fragment-13pf	Fragment-14pf	Fragment-13pf	13pf
Stabilizing molecule	MAP7 MTBD	Taxol	Taxol	Peloruside	Peloruside
Decorating protein	MAP7 MTBD	MAP7 MTBD	MAP7 MTBD	MAP7- FL	MAP7 MTBD and tau

Supplementary Fig. 7: Comparison of cryo-EM structures of MAP7 MTBD bound to MTs. Segments of cryo-EM density maps corresponding to the entire MAP7 MTBD bound to four tubulin monomers from various 3D reconstructions of MAP7 MTBD-MT complexes are shown in the top panel with luminal views of the corresponding central tubulin heterodimer shown in the bottom panel. Density for the α-tubulin, β-tubulin and MAP7 MTBD is shown in light grey, dark grey and teal respectively for maps determined in this study and green, blue and purple respectively for structures determined in [1]. Density corresponding to bound taxol molecule is shown in yellow and tau is shown in pink.

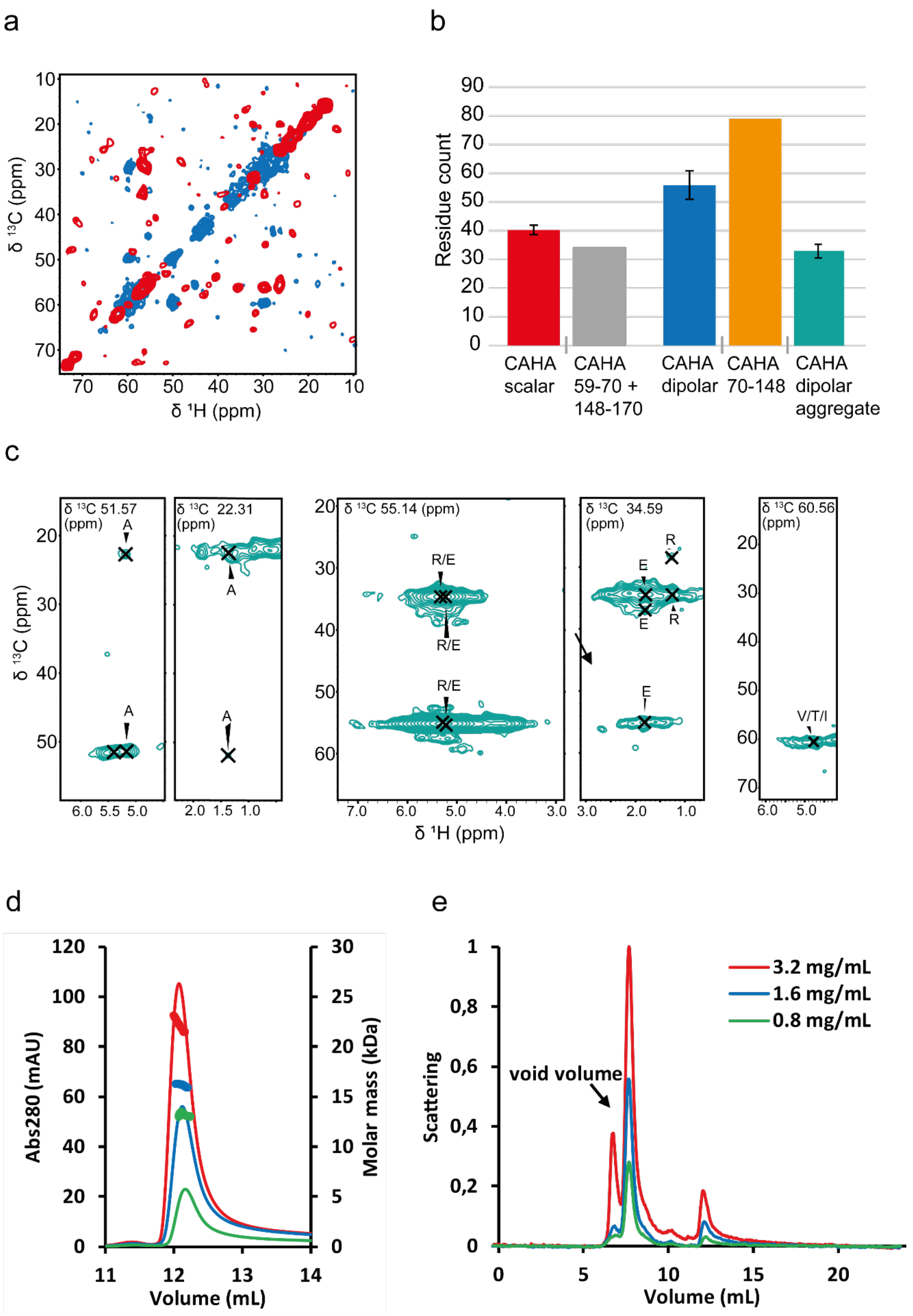
a
b
c
d
e
δ ¹³C (ppm)
δ ¹H (ppm)
Residue count
CAHA scalar
CAHA 59-70 + 148-170
CAHA dipolar
CAHA 70-148
CAHA dipolar aggregate
δ ¹³C 51.57 (ppm)
δ ¹³C 22.31 (ppm)
δ ¹³C 55.14 (ppm)
δ ¹³C 34.59 (ppm)
δ ¹³C 60.56 (ppm)
A
R/E
R
E
V/T/I
Abs280 (mAU)
Molar mass (kDa)
Volume (mL)
Scattering
void volume
3.2 mg/mL
1.6 mg/mL
0.8 mg/mL

Supplementary Fig. 8: 3D CCH experiments of MAP7 MTBD in association with MT and concentration-dependent self-association of MAP7 MTBD in solution. a CC- planes of [^{13}C-^{15}N]-MAP7 MTBD bound to MT. Scalar and dipolar experiments are given in red and blue, respectively. **b** CAHA abundancy in MAP7 MTBD regions compared to peak integration of CAHA regions in the scalar and dipolar CH projections of the 3D CCH spectra. **c** Assigned strips of correlations in the dipolar 3D CCH that exhibit β-strand like chemical shifts. **d** SEC-MALS data at a range of MAP7 MTBD concentrations show a slight peak shift and an increase in determined molar mass. This indicates that the sample is heterogeneous under the peak and undergoes a concentration-dependent self-association. **e** Scattering traces for the SEC-MALS runs show the presence of aggregates. At the highest concentration, there are proportionally more aggregates in the void volume. Source data are provided as a Source Data file.

Supplementary Table 1: Assigned chemical shifts of MAP7 MTBD from CH solution-state NMR data on the basis of the CA, CB, HA and HB assignments deposited under BMRB accession number 51,730.

Assignment	^{13}C Shift	^{1}H Shift
P59CA-HA	63.03	4.35
P59CB-HB2	32.00	2.19
P59CB-HB3	32.00	1.77
V60CA-HA	62.29	3.96
V60CB-HB	32.76	1.93
V60CG1-HG1	21.14	0.83
L61CA-HA	54.88	4.32
L61CB-HB3	42.59	1.49
L61CD1-HD1	23.51	0.82
R62CA-HA	55.89	4.34
R62CB-HB2	31.01	1.79
R62CB-HB3	31.01	1.69
R62CD-HD	43.03	3.13
V63CA-HA	62.72	3.97
V63CB-HB	32.82	1.98
V63CG1-HG1	20.64	0.86
D64CA-HA	54.23	4.53
D64CB-HB2	41.24	2.63
D64CB-HB3	41.24	2.61
D65CA-HA	55.32	4.49
D65CB-HB2	41.23	2.62
R66CA-HA	58.36	4.01
R66CB-HB2	30.08	1.83
R66CD-HD	43.51	3.13
R66CG-HG	27.21	1.53
Q67CA-HA	57.56	4.13
Q67CB-HB2	28.71	2.04
R68CA-HA	58.26	4.03
R68CB-HB2	32.71	1.98
R68CB-HB3	32.71	1.78
L69CA-HA	56.56	4.09
L69CB-HB2	42.04	1.64
L69CB-HB3	42.04	1.49
L69CD1-HD1	23.36	0.80
A70CA-HA	54.04	4.09
A70CB-HB	18.58	1.37
R71CA-HA	58.10	4.06
R71CB-HB2	30.25	1.85
R71CB-HB3	30.25	1.84
R71CD-HD	43.49	3.02
E72CA-HA	58.23	4.07
E72CB-HB2	29.86	2.00
R74CA-HA	58.20	4.11
R74CB-HB2	30.32	1.76
R74CB-HB3	30.32	1.54
R74CD-HD	43.35	3.10
E75CA-HA	58.37	4.08
E75CB-HB2	30.32	2.02
E75CB-HB3	30.32	1.83
E76CA-HA	58.40	4.10
E76CB-HB2	30.41	1.86
E76CB-HB3	30.41	1.82
K79CA-HA	58.15	4.11
K79CB-HB2	32.67	2.05
K79CB-HB3	32.67	1.83
K79CG-HG	25.05	1.50
Q80CA-HA	57.16	4.14
E100CA-HA	58.28	4.07
K101CA-HA	58.37	4.09
K101CB-HB2	31.00	1.84
K101CB-HB3	31.00	1.78
E104CA-HA	58.81	4.08
E104CB-HB2	29.57	1.86
E104CB-HB3	29.57	1.83
E105CA-HA	58.32	4.08
E105CB-HB2	29.77	2.05
E105CB-HB3	29.77	2.01
R106CA-HA	58.99	3.92
K107CB-HB2	32.51	1.84
E111CB-HB2	29.47	1.99
E111CB-HB3	29.47	1.85
E112CA-HA	59.03	4.04
E112CB-HB3	29.46	1.84
Q113CA-HA	58.56	4.14
Q113CB-HB2	28.49	1.98
Q113CB-HB3	28.49	1.79
E118CA-HA	58.79	4.04
R119CA-HA	58.79	4.44
R119CB-HB2	30.22	1.85
R120CB-HB2	30.24	1.85
R120CB-HB3	30.24	1.78
R121CA-HA	58.81	3.97
A122CA-HA	54.20	4.13
A123CA-HA	54.29	4.18
A123CB-HB	18.35	1.38
V124CA-HA	65.48	3.71
V124CB-HB	32.19	1.83
V124CG2-HG2	22.13	1.01
E125CG1-HG1	36.13	2.24
R128CA-HA	58.28	4.08
R128CB-HB2	29.65	1.97
R128CB-HB3	29.65	1.80
R129CA-HA	57.38	4.08
Q130CA-HA	57.60	4.21
Q130CB-HB2	28.78	1.42
Q130CB-HB3	28.78	1.37
L132CA-HA	56.73	4.07
L132CD1-HD1	24.58	0.78
E134CA-HA	57.91	4.10
E134CB-HB2	30.05	2.06
E134CB-HB3	30.05	2.01
H139CA-HA	57.16	4.45
H139CB-HB2	30.05	3.16
H139CB-HB3	30.05	3.11
E140CA-HA	57.45	4.05
A141CA-HA	53.47	4.14
A141CB-HB	18.89	1.36
V142CA-HA	63.71	3.82
V142CB-HB	32.45	2.04
V142CG1-HG1	20.79	0.87
V142CG2-HG2	21.28	0.91
V143CA-HA	63.67	3.81
V143CB-HB	32.34	1.94
R145CA-HA	56.99	4.24

Q80CB-HB2	28.79	2.05
Q80CB-HB3	28.79	1.83
L81CA-HA	56.40	4.11
L81CB-HB2	42.15	1.66
L81CB-HB3	42.15	1.50
L81CG-HG	27.25	1.60
A82CA-HA	53.28	4.15
A82CB-HB	18.82	1.37
A83CA-HA	53.35	4.15
A83CB-HB	18.83	1.36
R84CA-HA	57.05	4.05
R84CB-HB2	30.63	1.98
E85CA-HA	57.38	4.15
E85CB-HB2	30.11	1.97
E85CG-HG	36.50	2.31
I86CA-HA	62.34	3.90
I86CB-HB	38.36	1.77
I86CD1-HD1	12.78	0.76
I86CG1-HG1	27.92	1.10
I86CG2-HG2	17.25	0.67
V87CA-HA	63.65	3.90
V87CB-HB	32.32	1.95
V87CG1-HG1	20.88	0.83
W88CA-HA	58.45	4.46
W88CB-HB2	29.42	3.29
W88CB-HB3	29.42	3.18
L89CA-HA	56.38	4.07
L89CB-HB3	42.48	1.48
E90CA-HA	58.04	4.03
E90CB-HB2	29.78	1.99
E90CB-HB3	29.78	1.85
R91CA-HA	58.15	4.02
R91CB-HB2	30.41	1.98
R91CB-HB3	30.41	1.80
E92CB-HB2	29.70	1.95
E93CB-HB3	29.68	1.83
R94CA-HA	56.73	4.26
R94CB-HB2	30.25	1.78
R94CB-HB3	30.25	1.48
A95CA-HA	54.01	4.09
A95CB-HB	18.85	1.40
R96CA-HA	58.18	4.02
R96CB-HB2	30.24	2.00
R96CB-HB3	30.24	1.85
Q97CA-HA	58.23	4.09
H98CA-HA	57.70	4.11
H98CB-HB2	29.89	3.12
Y99CA-HA	59.84	4.32
Y99CB-HB2	38.44	3.07

R145CB-HB2	30.70	1.80
R145CB-HB3	30.70	1.76
R145CD-HD	43.46	3.18
T146CB-HB	69.57	4.18
M147CB-HB2	32.83	1.99
M147CE-HE	16.90	2.00
E148CG1-HG1	36.20	2.20
R149CA-HA	56.48	4.24
R149CB-HB2	30.69	1.82
R149CB-HB3	30.69	1.73
S150CB-HB3	63.82	3.81
Q151CA-HA	55.46	4.28
Q151CB-HB2	29.66	2.03
Q151CB-HB3	29.66	1.89
P153CA-HA	63.14	4.32
P153CB-HB2	32.18	2.21
P153CB-HB3	32.18	1.79
P153CD-HD	50.41	3.57
P153CG-HG	27.32	1.94
K154CA-HA	56.49	4.15
K154CB-HB	32.98	1.68
K154CG-HG	24.87	1.35
Q155CA-HA	55.53	4.21
Q155CB-HB2	29.78	1.91
Q155CB-HB3	29.78	1.84
N158CA-HA	53.15	4.52
N158CB-HB3	38.81	2.58
R159CA-HA	56.61	4.03
R159CB-HB2	30.43	1.85
R159CB-HB3	30.43	1.49
R159CD-HD	43.13	2.89
R159CG-HG	26.65	1.20
W160CA-HA	57.20	4.56
W160CB-HB3	29.53	3.06
S161CB-HB2	63.75	3.57
W162CA-HA	57.51	4.52
W162CB-HB2	29.55	3.17
W162CB-HB3	29.55	3.13
G163CA-HA	45.58	3.77
G164CA-HA	45.24	3.76
S165CB-HB2	62.98	4.16
H167CA-HA	55.65	4.53
H167CB-HB2	29.89	3.08
H167CB-HB3	30.16	2.94
G168CA-HA	45.17	3.83
S169CB-HB3	63.47	3.71
P170CA-HA	64.90	4.15
P170CD-HD	50.56	3.73

Supplementary Table 2: Assigned chemical shifts of the MAP7 MTBD in solid-state scalar and dipolar CCH spectra. Resonance assignments utilized for the reference peaks of the residue abundance estimation in Fig. 5 are highlighted in bold. Resonances that were assigned to residue types are given by letters instead of numbers in line with the nomenclature used in Figures 3 and 4.

Dipolar CCH		
**Residue**	**Atom**	**Shift**
**Aa**	CA	50.25
**Aa**	CB	14.96
**Aa**	HA	4.57
**Ab**	CA	54.80
**Ab**	CB	18.23
**Ab**	HA	4.05
**Ab**	HB	1.33
**Ac**	**CA**	**54.91**
**Ac**	CB	19.37
**Ac**	**HA**	**4.43**
**Ea**	CA	59.72
**Ea**	CB	29.59
**Ea**	CG	36.53
**Ea**	HB2	2.28
**Ea**	HA	3.98
**Ea**	HG2	1.93
**Eb**	CB	31.06
**Eb**	CG	36.70
**Eb**	HG2	2.10
**Eb**	CB	30.32
**Eb**	CG	34.26
**Eb**	HG2	1.25
**Eb**	HA	3.78
**Ka**	CA	59.66
**Ka**	CB	31.69
**Ka**	CD	29.44
**Ka**	CE	43.06
**Ka**	CG	25.21
**Ka**	HD2	1.57
**Ka**	HA	3.78
**Ka**	HG2	1.86
**Ka**	HB2	1.57
**Ka**	HE2	1.70
**La**	CD2	22.42
**La**	CG1	28.56
**La**	CD1	25.92
**La**	HD1	0.93
**La**	HD2	0.73
**La**	HG	0.80
**Lb**	CB	42.08
**Lb**	HB	1.57
**Lc**	CD	25.49
**Lc**	HD1	0.79
**Lc**	HD2	0.82
**Ld**	**CB**	**42.24**
**Ld**	**HB**	**1.4**
**Ma**	CB	36.53
**Ma**	CE	20.61

Scalar CCH		
**Residue**	**Atom**	**Shift**
**Ea**	CA	56.3
**Ea**	CG	35.7
**Ea**	CB	29.62
**Ea**	HA	4.1
**Ea**	HB2	2.16
**Ea**	HG3	1.96
**E/Ra**	CA	55.75
**E/Ra**	CB	30.23
**E/Ra**	HA	4.23
**La**	CD2	23.17
**La**	HD2	0.83
**P/V/Ka**	CB	32.67
**P/V/Ka**	HB3	1.74
**Qa**	CA	55.22
**Qa**	HA	3.9
**R/La**	CA	55.93
**R/La**	CD	42.12
**R/La**	CG	26.5
**R/La**	HG3	1.59
**Sa**	CB	62.28
**Sa**	HB3	3.51
**La**	CD2	23.24
**La**	HD2	0.79
**Ra**	CA	56.2
**Ra**	CB	30.41
**Ra**	CG	26.49
**Ra**	HG2	1.78
**P59**	CA	62.28
**P59**	CB	31.6
**P59**	HA	4.33
**V60**	CA	62.07
**V60**	CB	31.73
**V60**	CG1	20.41
**V60**	CG2	20.26
**V60**	HA	3.95
**V60**	HB	1.97
**V60**	HG1	0.85
**L61**	CA	54.35
**L61**	CB	42.13
**L61**	CD2	22.84
**L61**	CG	26.06
**L61**	HB	1.61
**L61**	HG	1.51
**D64**	CA	53.98
**D64**	CB	40.42
**D64**	HA	4.47
**D64**	HB	2.61
**L69**	CA	55.34

**Ma**	HB2	2.20
**Ma**	HE	1.92
**Qa**	CA	59.84
**Qa**	CB	29.21
**Qa**	CG	32.22
**Qa**	HB2	1.88
**Qa**	HA	4.07
**Qa**	HG2	1.69
**Ra**	CA	57.01
**Ra**	CB	29.62
**Ra**	CD	42.98
**Ra**	CG	27.91
**Ra**	HD2	3.11
**Ra**	HB2	1.50
**Ra**	HG2	1.58
**Rb**	CG	28.20
**Rb**	CA	59.45
**Rb**	HD2	3.11
**Rb**	HB2	1.65
**Rc**	HG2	1.58
**Sa**	CA	59.93
**Sa**	**CB**	**61.97**
**Sa**	**HB2**	**4.18**
**Sb**	CA	59.84
**Sb**	CB	62.21
**Sb**	HB2	4.01
**Va**	CB	31.94
**Va**	CG	21.47
**Va**	HG1	1.16
**Vb**	**CG**	**22.78**
**Vb**	**HG1**	**0.99**

**L69**	CB	42.17
**L69**	CD1	26.36
**L69**	CD2	23.04
**L69**	CG	26.42
**L69**	HA	4.17
**L69**	HD1	1.57
**L69**	HD2	0.77
**L69**	HG	1.56
**I86**	**CD1**	**11.81**
**I86**	CD2	22.5
**I86**	CG2	12.15
**I86**	**HD1**	**0.75**
**I86**	HG2	0.81
**T146**	CA	62.47
**T146**	**CB**	**70.36**
**T146**	CG2	20.96
**T146**	HA	4.12
**T146**	**HB**	**4.09**
**T146**	HG2	1.1
**M147**	CA	60.15
**M147**	CE	16.42
**M147**	HE	1.92
**E148**	CA	56.42
**E148**	CB	29.56
**E148**	CG	35.69
**E148**	HA	4.12
**E148**	HG3	1.95
**K154**	CD	28.39
**K154**	CG	24.15
**K154**	HD3	1.58
**K154**	HG3	1.34
**Q155**	CA	55.27
**Q155**	CB	30.38
**Q155**	CG	33.32
**Q155**	HA	3.89
**Q155**	HG3	2.2
**N158**	CA	53.54
**N158**	**CB**	**38.5**
**N158**	HA	4.57
**N158**	**HB**	**2.59**
**R159**	CA	56.11
**R159**	CB	30.37
**R159**	**CD**	**42.69**
**R159**	CG	26.87
**R159**	HA	4.16
**R159**	HB2	2.22
**R159**	**HD2**	**3.1**
**R159**	HG2	1.71
**L166**	CB	41.01
**L166**	CD2	23.85
**L166**	HD2	0.82
**P170**	CA	64.86
**P170**	CB	31.96
**P170**	HA	4.18
**P170**	HB2	2.13

Supplementary references

1. Ferro LS*, et al.* Structural and functional insight into regulation of kinesin-1 by microtubule-associated protein MAP7. *Science* **375**, 326–331 (2022).
